# Cell migration on microposts with surface coating and confinement

**DOI:** 10.1042/BSR20181596

**Published:** 2019-02-19

**Authors:** Jianan Hui, Stella W. Pang

**Affiliations:** 1 Department of Electronic Engineering, City University of Hong Kong, Hong Kong, China; 2 Center for Biosystems, Neuroscience, and Nanotechnology, City University of Hong Kong, Hong Kong, China

**Keywords:** Cell migration, cell confinement, cell deformation, surface coating, three-dimensional microenvironment

## Abstract

Understanding cell migration in a 3D microenvironment is essential as most cells encounter complex 3D extracellular matrix (ECM) *in vivo*. Although interactions between cells and ECM have been studied previously on 2D surfaces, cell migration studies in 3D environment are still limited. To investigate cell migration under various degrees of confinements and coating conditions, 3D platforms with micropost arrays and controlled fibronectin (FN) protein coating were developed. MC3T3-E1 cells spread and contacted the top surface of microposts if FN was coated on top. When FN was coated all over the microposts, cells were trapped between microposts with 3 μm spacing and barely moved. As the spacing between microposts increased from 3 to 5 μm, cells became elongated with limited cell movement of 0.18 μm/min, slower than the cell migration speed of 0.40 μm/min when cells moved on top. When cells were trapped in between the microposts, cell nuclei were distorted and actin filaments formed along the sidewalls of microposts. With the addition of a top cover to introduce cell confinement, the cell migration speed was 0.23 and 0.84 μm/min when the channel height was reduced from 20 to 10 μm, respectively. Cell traction force was monitored at on the top and bottom microposts with 10 μm channel height. These results show that the MC3T3-E1 cell morphology, migration speed, and movement position were affected by surface coating and physical confinement, which will provide significant insights for *in vivo* cell migration within a 3D ECM.

## Introduction

Cells encounter complex 3D extracellular matrix (ECM) *in vivo*. Movement of cells is affected by the ECM surface properties and physical confinement [1–3]. For example, the collective migration of epithelial and endothelial cells in capillary-like 3D structures initiates tissue development [4]. Neutrophil cells need to squeeze through cellular layers to enter the inflammatory area [5]. Cancer metastasis involves cancer cell invasion through cellular sheets and basement membrane [6].

Various studies have investigated the cell–ECM interactions on 2D surfaces including traction force generation [7], focal adhesion area [8], and myosin-based cytoskeletal mechanics [9]. However, cells behave differently during migration in a 3D confined microenvironment. For example, the migration mode of cells switched from mesenchymal to amoeboid fashion as cells migrated in highly confined and low-adhesive ECM [10]. Therefore, different cell migration mechanisms are expected as cells move in 3D confined ECM compared with 2D flat surface [2,11].

During 3D cell migration, the pulling force built up between integrin-dependent focal adhesions and actin filaments is essential for extending lamellipodia through narrow gaps and moving a cell forward [12]. Also, the flexibility of cell nucleus to change its shape in confinement was reported to be the rate limiting factor for 3D cell migration through tight enclosures because cell nucleus is the most rigid cellular organelle [3,5]. The actin-filament generated force has been reported to deform nucleus by linker of the nucleoskeleton and cytoskeleton (LINC) complexes [13–15]. Hence the development and correlations between cell nucleus, actin-filament distribution, and focal adhesions are essential for cell migration in 3D confined microenvironment and yet they are still poorly understood.

On the other hand, 3D ECM *in vivo* consists of various physical dimensions, surface properties, and stiffness, which is very different from a homogeneous 2D surface [16]. Collagen matrices have been widely used to create 3D fibrous matrix with different pore sizes and stiffness by controlling the concentration and cross-linking temperature [12]. Other 3D structures, such as curved structures [17] and wells [18] have been developed to mimic the 3D matrix and study the cellular responses under various geometries. Confined channels [19] and micropost arrays [20,21] have been fabricated to study cell migration under confinement smaller than the cell size. All these results provided useful information related to cellular interactions in 3D. However, these studies have some limitations. The porosity of 3D collagen matrix, which is used to provide the confinement effect, is controlled by the concentration and polymerization temperature of the collagen fiber [12]. However, the porosity and stiffness cannot be controlled precisely. Hence, studying the effects of 3D topography with precise dimensions and stiffness is difficult when collagen is used as a substrate. In addition, very low porosity of hydrogels precludes the study of 3D cell migration on a loose matrix [22]. On the other hand, stiffness of microposts was controlled by material properties and post dimensions, including diameter (dia.) and height of the polydimethylsiloxane (PDMS) posts. Previous studies using microposts focussed on cell spreading and migration when cells contacted only the top surface of microposts, which represented cell migration behavior on a 2D flat surface [7,23]. In this study, by controlling the coating conditions and integrating a top cover, the micropost platforms could be used to study 3D cell migration under various degrees of confinements.

In the present study, microfabricated post arrays were integrated with channels to create the microenvironment with various degrees of confinement and different surface coatings. When cells migrated under different micropost spacing and coating conditions, cell motility and trajectories were investigated and correlated with nucleus deformation, cytoskeleton distribution, and cell spreading using time-lapse images. The cell morphology, migration speed, and directionality were largely affected by the spacing between microposts. Various degrees of confinement and surface coating conditions influenced cell spreading and movement position in the 3D platforms. Understanding cell migration in 3D ECM will be useful for designing *in vitro* platforms to selectively control cell migration in a biomimetic microenvironment.

## Materials and methods

### Microfabrication technology and surface functionalization of PDMS platforms

**Figure 1:**
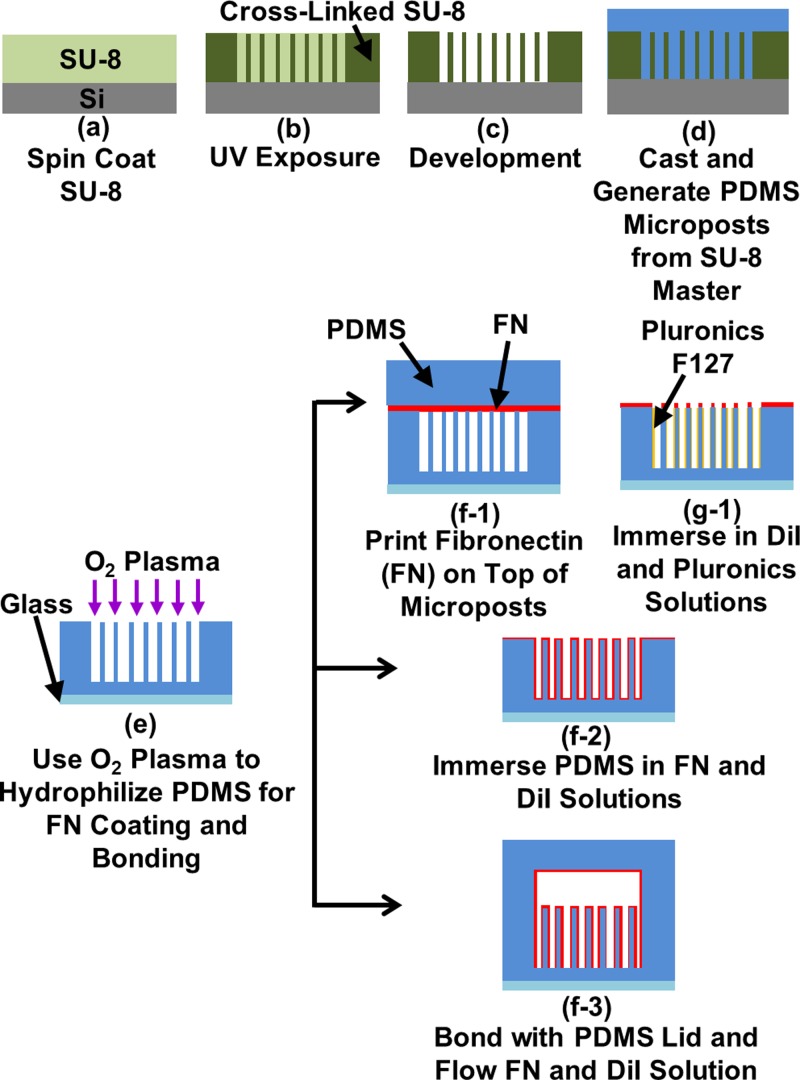
Fabrication technology for creating cell migration platforms with different coatings and confinements (**a**-**e**) Replicating polydimethylsiloxane (PDMS) microposts from SU-8 master molds and using oxygen plasma for hydrophilic surface. (**f-1, g-1**) Coating fibronectin (FN) on top of microposts while blocking cellular contact on sidewalls. (**f-2**) Coating all over microposts. (**f-3**) Adding cover on top of microposts for confinement.

PDMS platforms were replicated from SU-8 master molds, as shown in Figure 1(a–d). SU-8 (Microchem, MA, U.S.A.) master molds were patterned by UV lithography and treated with trichloro(^1^H,^1^H,^2^H,^2^H-perfluorooctyl)silane (FOTS) (Sigma–Aldrich, WI, U.S.A.) to form an anti-sticking layer. To create the microposts inside a confined channel, two layers of SU-8 were spin-coated and exposed twice sequentially followed by a single development, similar to previous work [23]. PDMS prepolymer (base monomer:curing agent weight ratio = 10:1, Sylgard 184, Dow Corning, MI, U.S.A.) was poured on to the SU-8 master mold to generate a soft PDMS mold. The PDMS micropost platform was generated by casting on a soft PDMS mold and cured under a 110°C convection oven for 6 h. After peeling off from the soft mold, collapsed PDMS microposts was ultra-sonicated in absolute ethanol (≥99.8%, Sigma–Aldrich, WI, U.S.A.) so that the tall posts could be separated and supercritically dried in a critical point dryer (EM CPD300, Leica, Hesse, Germany).

**Figure 2:**
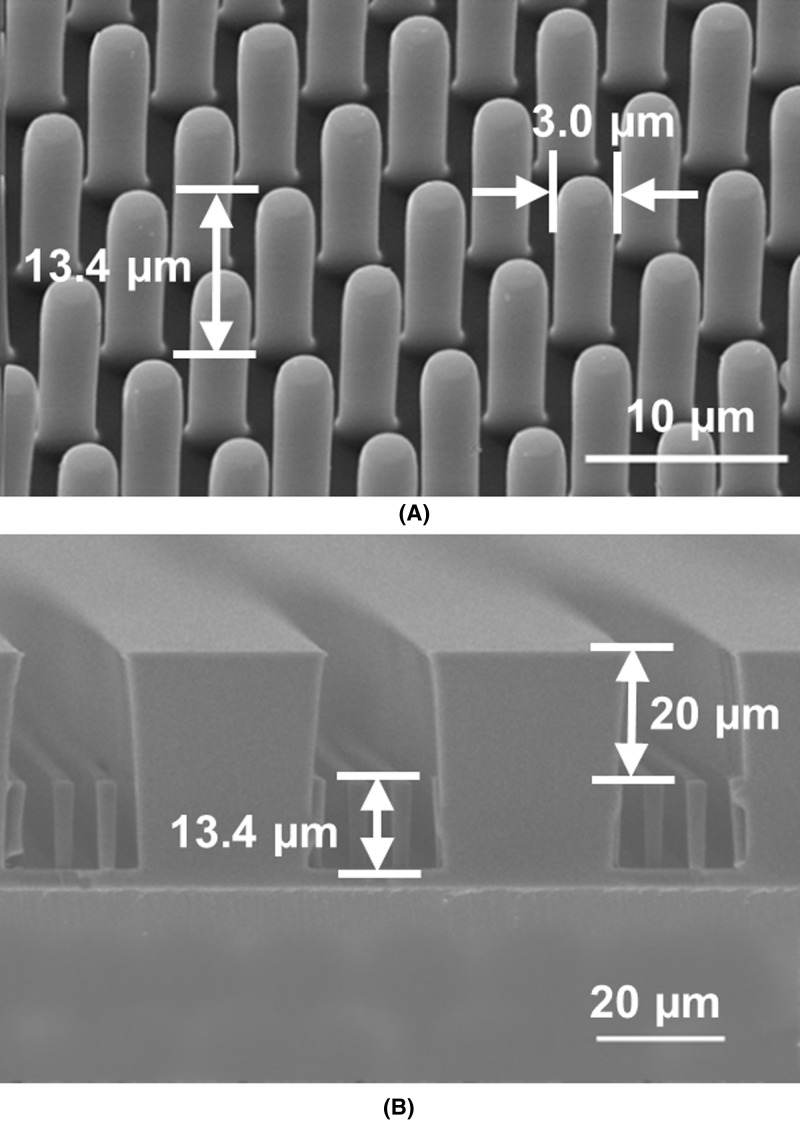
Scanning electron micrographs of fabricated cell migration platforms with microposts (**A**) Micrograph of fabricated PDMS microposts, 3 μm dia. and 13.4 μm height. (**B**) Fabricated PDMS micropost arrays with additional 20-μm thick PDMS separation from cover.

To coat ECM protein on these micropost platforms, the microposts were hydrophilized by a microwave ashing plasma system (GIGAbatch 310 M, PVA TePla, Wettenberg, Germany) with the following conditions: 135 sccm O_2_, 15 sccm N_2_, 150 mTorr, and 30 W rf power within Faraday cage for 15 s, as shown in Figure 1(e). Contact printing was used to coat fibronectin (FN, 50 μg/ml in deionized water, Sigma–Aldrich, MO, U.S.A.) on top of the microposts, as shown in Figure 1(f-1). To prevent cell adhesions on the sidewalls of microposts, the micropost platform was immersed in 0.2% Pluronic F-127 (Sigma–Aldrich, WI, U.S.A.) [24], as shown in Figure 1(g-1). Coating FN on top of the microposts would keep the cell movement on top and not to be trapped in between the microposts [25,26]. In comparison, the hydrophilized PDMS micropost platform was immersed in 50 μg/ml FN solution for 3 h to coat protein all over the microposts, as shown in Figure 1(f-2). In this case, the cells could spread in between the microposts under tightly confined 3D environment. To label the microposts for high-contrast images to capture the displacement of the posts, the micropost platforms were submerged in lipophilic dye, DiI (5 μg/ml in distilled water, 1,10-dioleyl-3,3,30,30-tetramethylindocarbocyanine methanesulphonate, Invitrogen, CA, U.S.A.) for 1 h and then rinsed with PBS to remove excess DiI molecules before cell seeding.

**Figure 3:**
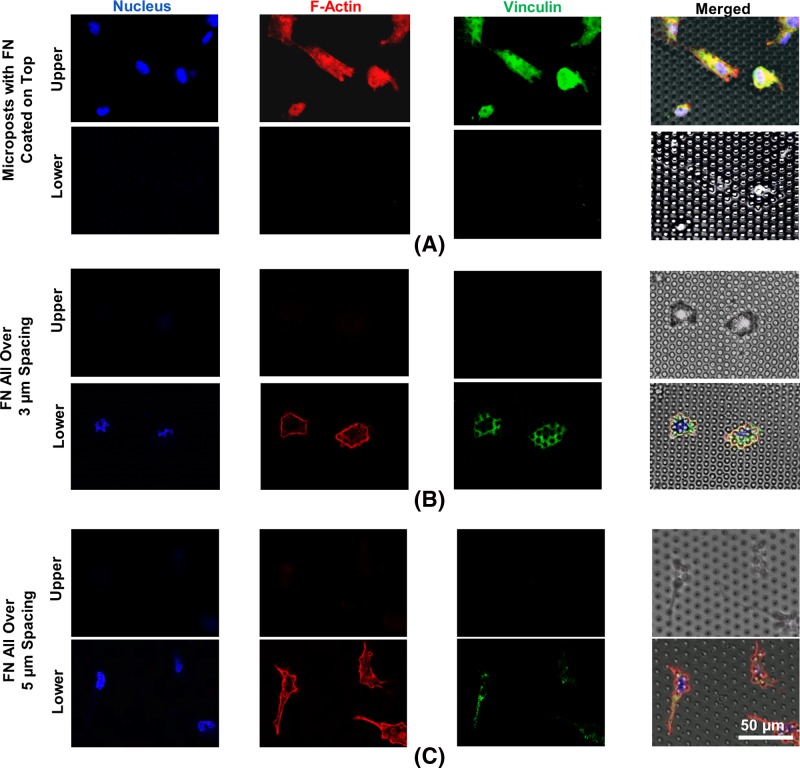
Immunofluorescent micrographs of cells cultured on FN coated microposts (**A**) FN coated on top with 5 μm spacing. (**B**) 3 μm and (**C**) 5 μm spacing microposts immersed in FN solution. All microposts were 3 μm in dia. Blue, nucleus; red, F-Actin; and green, vinculin.

Confined 3D platforms containing the microposts at the bottom and a cover plate on top were generated by bonding two layers of PDMS together. An O_2_ plasma with 1350 sccm O_2_, 150 sccm N_2_, 750 mTorr, and 50 W rf power for 15 s was used to increase the silanol groups (Si–OH) on the PDMS surfaces so that they form strong covalent bonds (Si–O–Si) when the two PDMS layers were brought together [27]. FN solution (50 μg/ml) was injected into the platform with confined channel and incubated for 3 h. Excess FN was purged by flushing with PBS in the microfluidic channel. Then, 5 μg/ml DiI was flown through the microfluidic channel for 1 h and PBS was applied to remove excess DiI, as shown in Figure 1(f-3). Before cell seeding, fresh culture medium, described in detail in the following section, was flushed through the microfluidic channel to replace PBS.

### Cell culture and seeding on PDMS platforms

MC3T3-E1 osteoblastic cells were obtained from American Type Culture Collection (ATCC numbers CRL-2594) and maintained in the cell culture medium, Dulbecco’s modified Eagle’s medium (DMEM), at 37°C and 5% CO_2_. The DMEM consisted of high glucose (Invitrogen, CA, U.S.A.) with 10% FBS (Gibco, MD, U.S.A.), antibiotic-antimycotic (100 units/ml of penicillin, 100 mg/ml of streptomycin, and 0.25 mg/ml of Amphotericin B, Gibco, MD, U.S.A.), and 2 mM alanyl-l-glutamine (Gibco, MD, U.S.A.). The cells were kept below full confluence at all times. The MC3T3-E1 cells were trypsinized (0.05% w/v trypsin in EDTA) and seeded at a density of 5 × 10^3^ cells/ml on the open micropost platforms. For seeding MC3T3-E1 cells in the confined micropost platform with a top cover, cells at a density of 1 × 10^5^ cells/ml in DMEM were delivered into the channel by a syringe. The culture dish was maintained at 37°C and 5% CO_2_ for 6 h to allow the complete attachment of cells on to the PDMS platform. The DMEM was then replaced by a CO_2_-independent medium (Invitrogen 18045-088, CA, U.S.A.), 10% FBS, antibiotic-antimycotic, and supplemented with 2 mM alanyl-l-glutamine (Gibco, MD, U.S.A.) for time-lapse imaging. The MC3T3 cell passage was controlled in the range of 3–20.

### Cell migration observed under time-lapse confocal microscope

A laser scanning confocal microscope (TCS SP5, Leica, Hesse, Germany) was used to take time-lapse images at a time interval of 1 min for 6 h. Four adjacent positions were chosen to observe multiple cells. To maintain the focus over long imaging time, the stage movement was controlled within 1 mm. A 40× oil immersion objective lens was used for imaging single cell movement and micropost bending under high magnification. To acquire the bending of the PDMS posts, the top of the bottom positions of the DiI-labeled microposts were determined by changing the focussing position through a 80-μm pinhole. To keep track of the cell migration paths, time-lapse images in bright field were recorded using a 20× magnification lens.

### Immunofluorescence staining and confocal imaging

To fix MC3T3-E1 cells on the open micropost platform, cells were cultured on PDMS platform 6 h and then submerged in 4% (w/v) paraformaldehyde (PFA, Sigma–Aldrich, WI, U.S.A.) in PBS for 15 min at room temperature. After washing the excess PFA with PBS, cells were permeabilized with 0.1% Triton X-100 in PBS for 5 min. The PDMS platform was then washed with PBS and soaked in blocking solution (1% BSA in PBS) for 30 min to eliminate non-specific binding. The cells were first incubated with primary antibody mouse anti-vinculin (Millipore, MA, U.S.A.) for 80 min and rinsed in PBS. Then the cells were incubated with secondary antibody (goat anti-mouse, fluorochrome–conjugated Alexa 488, Invitrogen, CA, U.S.A.) to stain vinculin. A 0.165 M rhodamine-phalloidin (Invitrogen, CA, U.S.A.) was incubated together with second antibody for 1 h at room temperature to label actin filaments simultaneously. After washing with PBS, the cells were stained with DAPI (300 nM, Millipore, MA, U.S.A.) in a blocking solution for 10 min for visualization of cell nucleus.

Cells were imaged using a confocal microscope (TCS SPE, Leica, Hesse, Germany) with 63× oil immersion objective lens (HCX APO 63×/1.4-0.60, Leica, Hesse, Germany). To get the fluorescent signals of cells along the sidewalls of microposts, scanned images from the bottom to top of the microposts were obtained through a 80-μm pinhole. The resolution along the micropost sidewall could be improved by setting the size of the pin hole to be comparable with the dia. of Airy disc:

(1) $$d_{\text{Airy}}\;=\;\frac{1.22\lambda }{NA}\cdot M\cdot 3.6$$

where *λ* is the excitation wavelength, *NA* is the numerical aperture, *M* is the magnification of the lens, and the factor 3.6 refers to the magnification brought by other optical components in the Leica confocal system. Using eqn 1, the calculated dia. of Airy disc is 75 μm for DAPI and 107 μm for rhodamine-phalloidin. Therefore, the optical images with both high contract and brightness can be obtained by setting the pinhole size to 80 μm.

### Mechanical properties of micropost platforms

A field-emission scanning electron microscope (FE-SEM, SU5000, Hitachi, Tokyo, Japan) was used to measure the dimensions of the fabricated microposts and confined channel, as shown in Figure 2. The microposts were 13.4 μm tall with 3 μm dia., and they were arranged in hexagonal arrays as shown in Figure 2A. The spacing from edge to edge between two adjacent microposts was varied from 3 to 5 μm in order to study the effect of confinement. The PDMS showed a Young’s modulus of 2.5 MPa after curing in 110°C for 6 ± 0.5 h. Figure 2B shows a cross-section of the micropost platform with 20 μm channel height. A tighter confinement of 10 μm was also produced to study the effects of channel confinement on cell migration.

### SEM showing cell morphology

For observation of the cells under SEM, the cells were first fixed after 6 h seeding on the PDMS platform, which was described in ‘Immunofluorescence staining and confocal imaging’ section. The cells were rinsed with PBS at 37°C, followed by fixation with 4% PFA for 15 min. After thoroughly rinsing with PBS, the PDMS platforms were dehydrated through an ascending concentration of ethanols (30, 50, 70, 80, 90, 95, and 100%). To reduce the artifacts created by surface tension, the cells were supercritically dried using a critical point dryer (EM CPD3000, Leica, Hesse, Germany). The PDMS platforms were then sputter-coated with gold.

### Data analysis

Cell movement was manually tracked by Manual Tracking plugin of NIH ImageJ software (version 1.48v). The strength of the fluorescent images was quantitated with similar methods mentioned in previous studies [28,29]. To avoid variation of absolute value of the fluorescent signal, the intensity was normalized by average value of each cell. Although the pinhole size was set to be comparable with the Airy unit as described in ‘Immunofluorescence staining and confocal imaging’ section, the fluorescent signal intensity was observed to be affected by absorption or scattering of PDMS and cell culture medium [30]. Signal decay on a flat surface without microposts was analyzed as shown in Supplementary Figure S1a. The signal intensity decreased substantially to 6.5% at 1.4 μm above the flat surface as the 80 μm pinhole blocked majority of the signal. The signal distribution of 15 microposts with no seeded cell was analyzed along the z-axis from the bottom to the top of microposts, as shown in Supplementary Figure S1b. Microposts with the height of 20 μm were fabricated and stained with DiI dye in a 5 μg/ml solution. The fluorescent signal should be uniform along the microposts but it showed a decreasing intensity due to the light scattering and geometric effect. This signal distribution was used to correct for the signal intensity of cell nucleus and actin filament from the bottom to the top of microposts.

To detect the bending of the microposts, a MATLAB (R2007b, The MathWorks, MA, U.S.A.) graphical user interface was used to process the images [25]. Fluorescent signals at the base and at top of the micropost were compared to deduce the lateral displacement at the top of the micropost. All the displacement results were presented with mean ± S.E.M. Statistical significance was tested by employing the Student’s *t* test and null hypothesis was rejected when *P*<0.05. At least three sets of runs were used for the analysis.

## Results and discussion

### Cell spreading during migration on top and in between microposts

Figure 3 shows the fluorescent signals of cell spreading on microposts with different FN coatings. To mimic cell movement in a 3D microenvironment, cell migration was compared between cells moving on top and in between microposts. To keep the cells on top, FN was coated on the top surface of microposts by contact-printing transferred FN to promote the cell adhesion, while the remaining regions were blocked with pluronics surfactants that resist protein absorption and cell adhesion. In addition, the spacing between the microposts was adjusted from 3 to 5 μm moved on top of microposts.

When FN was contact-printed on top of the microposts while the sidewalls were blocked with pluronics, strong fluorescent signals of cell nucleus, actin filament, and vinculin were observed on upper part of microposts, as shown in Figure 3A. Cell nucleus was observed to have a rounded shape in the cell center. The actin filaments were polymerized and generated a meshwork of filaments uniformly across the cellular body. The vinculin signal showed uniform distribution on top of the microposts. The immunofluorescent images of cell spreading on protein-coated microposts were similar to previous reports, where cells moved on top of microposts [31].

In comparison, when cells were seeded on microposts with FN coated all over, as shown in Figure 3B,C, fluorescent signals were observed along the sidewalls of the microposts. The cells were trapped or moved in between the microposts with 3 or 5 μm spacing. Cell nucleus was squeezed by the microposts and no longer had round shape. The F-actin was observed around the periphery of the cells and along the sidewalls of microposts. Similarly, the vinclulin signal was found around the microposts. The uneven distribution of actin-filament fluorophores across the cellular body was due to trapping of cells in the 3D-confined environment. Instead of having a sequential cycle consisting of the extension and attachment of the cell leading edge and retraction of the cell trailing edge in the direction of migration [23,32], the cells migrated within 3D microposts had more interactions with the vertical sidewalls along the microposts [20].

For microposts with FN coated all over, the cell morphology was more elongated with protruded lamellipodia between the microposts with 5 μm spacing. Stronger vinculin and F-actin signals were generated at elongated lamellipodia for FN coated around microposts with 5 μm spacing in Figure 3.

### Deformation of cell nucleus trapped within microposts

As tissue consists of dense fibrous extracellular network with pore size smaller than the size of cell nucleus, cells encounter tight confinement during migration in 3D ECM. Cells have to deform and squeeze through interstitial spaces in order to move around *in vivo*. As cell nucleus is large and rigid, the deformation of cell nucleus is considered to be the rate-limiting step during cell migration in 3D ECM [33].

**Figure 4:**
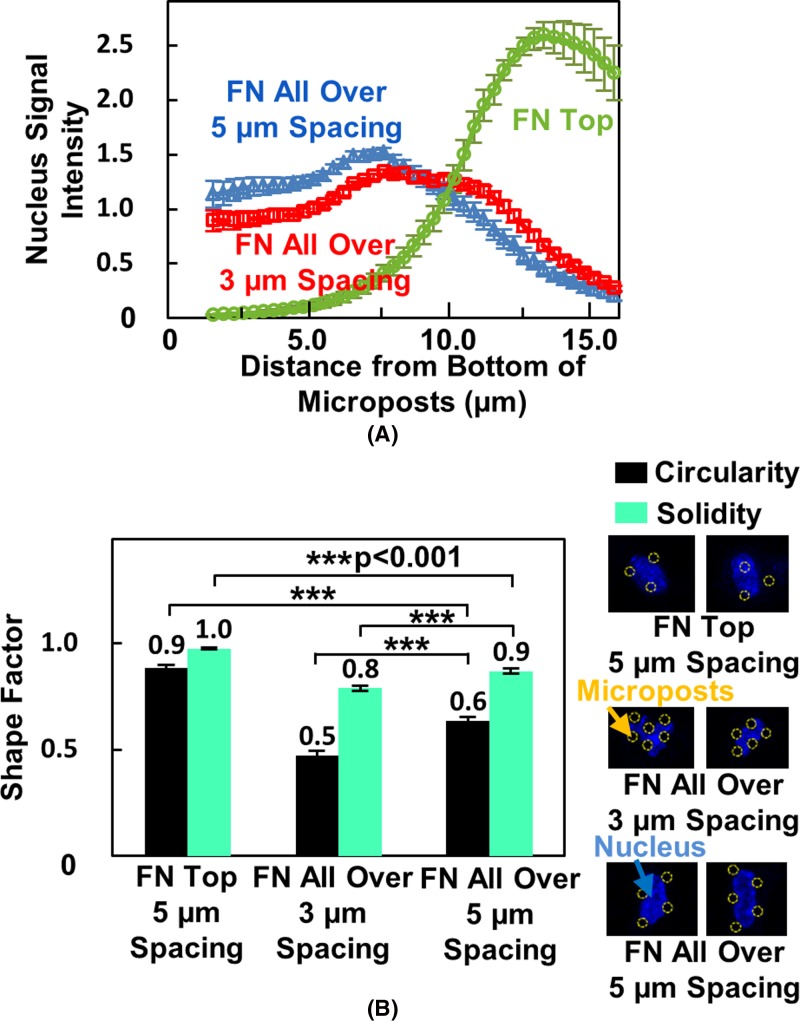
Position and deformation of cell nucleus on micropost platforms with different coatings and spacings (**A**) Cell nucleus signal intensity from bottom to top of microposts. (**B**) Circularity and solidity of nucleus. Cells were seeded on micropost platforms with FN coated on top or immersed in FN solution. Statistical significance was calculated using Student’s *t* test (***P<0.001).

The position of the cell nucleus was investigated by following the intensity of the fluorescent signal. When cell migrated on microposts with FN coating only on top, the cell nucleus fluorescent intensity was found to peak the average value and was located near the top of the microposts, as shown in Figure 4A. In this case, the cell nucleus stayed on the top surface of the microposts. However, for microposts with FN coated all over, the cell nucleus signal was found to distribute along the micropost sidewalls after 6 h seeding, as shown in Figure 4A. Therefore, the cells had to squeeze in between the vertical sidewalls of the microposts. As the cell nucleus was highly deformed by the sidewalls of the microposts, the ability for cell nucleus to deform and the morphology of the cell nucleus were related to cell migration [14,34].

Two shape factors, circularity and solidity, were taken into consideration [35]. The circularity, *C*, is related to how close the shape of cell nucleus to a circle.

(2) $$C\;=\;\frac{4\pi A}{P^{2}}$$

where *A* is the cell area and *P* is the perimeter of the cell nucleus. As shown in Figure 4B, the circularity of the cell nucleus was 0.88 ± 0.01 for cells on top of microposts with FN coated only on top, 0.47 ± 0.02 and 0.63 ± 0.02 for 3 and 5 μm spaced microposts with FN coated all over. This indicated that cell nucleus was able to deform into the limited spaces with confinement down to 3 μm, as indicated in the immunofluorescent-labeled nuclear images in Figure 4B. To further study the level of deformation, solidity, *S*, was used to indicate the overall concavity of the nucleus.

(3) $$S\;=\;\frac{A}{A_{c}}$$

where *A* is the cell nuclear area and *A_c_* is convex hull area. As nucleus was squeezed into the 3 and 5 μm spacing between microposts, the cell nucleus could change from a convex to a concave shape. The cell nucleus within the 3 μm spacing microposts showed a smaller solidity value of 0.79 ± 0.01 compared with 0.87 ± 0.01 for 5 μm spacing. This indicated that the cell nucleus was deformed into a more irregular shape when confined by microposts with 3 μm spacing. For cell migration on top of the microposts, cell nucleus was more rounded and it was not deformed by the microposts with a higher solidity of 0.97 ± 0.00. These results showed the differences of cell position and cell shape for cells migrating on top or in between microposts with various spacings.

### Development of cytoskeleton around cell periphery and along sidewalls of microposts

The deformation of cell nucleus is closely related to cytoskeletal network via LINC, which enabled force transmission between actin filament of cell nucleus to change nuclear shape [14,33]. Therefore it is useful to look into the cytoskeletal organization during cell spreading in 3D environment in order to understand 3D cell migration.

**Figure 5:**
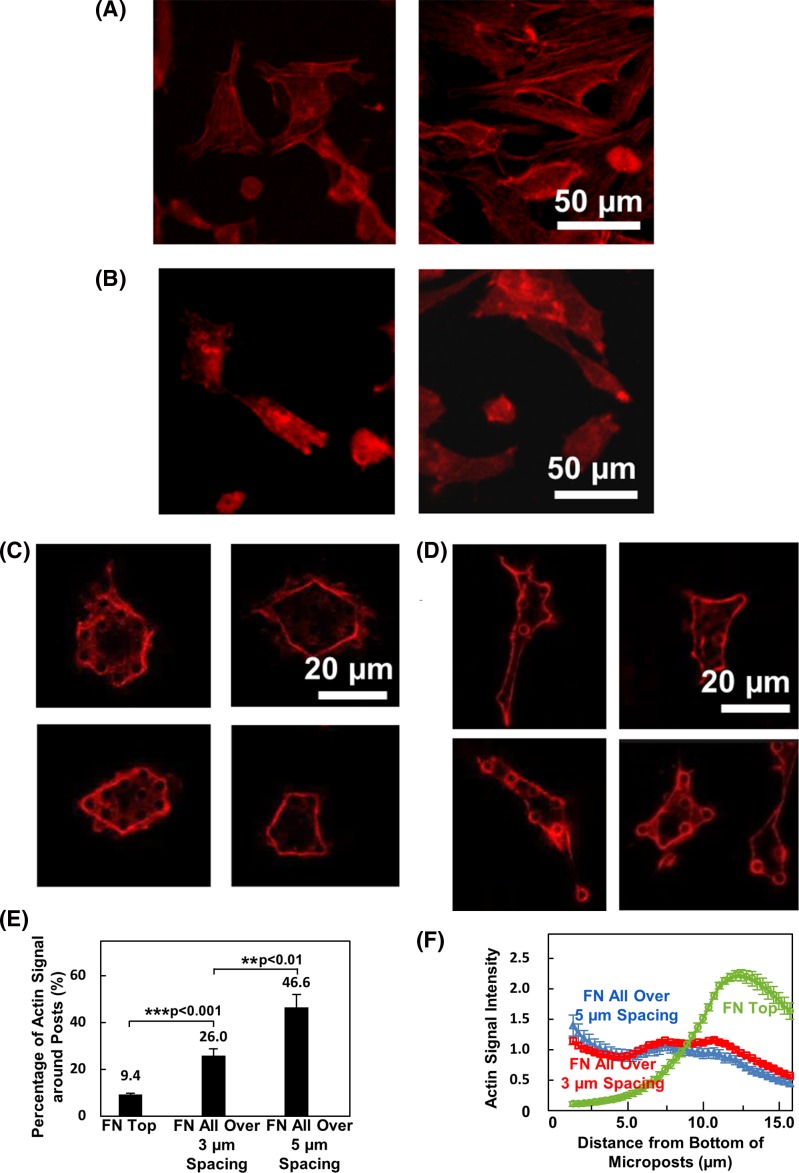
Actin-filament distribution on micropost platforms with different coatings and spacings Actin filament signal of cells seeded on (**A**) flat PDMS surface, (**B**) microposts with FN printed on top, (**C**) microposts with 3 μm spacing and FN coated all over, and (**D**) microposts with 5 μm spacing and FN coated all over. (**E**) Percentage of actin filament signal intensity around microposts. (**F**) Actin signal intensity from bottom to top of microposts. Statistical significance was calculated using Student’s *t* test (**P<0.01 and ***P<0.001).

As shown in Figure 5A, cells developed a meshwork of stress fibers that were uniformly distributed across cellular body during cell spreading on flat PDMS substrate. In 2D mesenchymal cell migration, the polarized extension of cell leading region and retraction of cell trailing region are driven by the continuous polymerization or depolymerization at cell edges. The meshwork of actin filament is essential for force engagement between actomyosin and focal adhesion, which contributes to dynamic movement of lamellipodia [36]. Similarly, cells developed bundles of actin filaments inside cells during cell migration on top of microposts, as shown in Figure 5B. This indicated a similar cell migration process when cells moved on top of microposts [31].

However, prominent actin filaments were developed only around cell periphery and along sidewalls of microposts during cell spreading and migration within microposts, as shown in Figure 5C,D. The strong signal of actin filament at cellular edges indicated a strong coupling between F-actin and ligand/receptor complexes at cell periphery, which could introduce a contracted cell shape [37]. The actin filaments also wrapped around sidewalls of microposts, similar to previous study showing actin stress fibers along microposts [20]. However, a stronger coupling between cytoskeleton and sidewalls of microposts was found for microposts with 5 μm spacing compared with 3 μm spacing. The actin-filament signal around the microposts was compared. As shown in Figure 5E, actin filament around microposts was 9.3 ± 0.4% when cell migrated on top of microposts. Majority of the cell F-actin was generated inside the cell body. A stronger coupling of actin and microposts was found for cells spreading on microposts with FN coated all over. The strongest signal of 46.6 ± 5.6% was found for cells spreading with 5 μm spacing while signal for cells in 3 μm spacing microposts was 26.0 ± 2.9%.

The actin distribution was also studied, as shown in Figure 5F. When cells migrated on top of the microposts, the cell F-actin intensity peaked near the top of the microposts. However, the actin-filament signal was found to distribute along the sidewalls of the microposts when cell migrated in between microposts with 3 or 5 μm spacing. As cell membrane was thin and flexible, the protrusions of the cytoskeleton could reach from top to bottom of the microposts. All these results have shown distinct organization and distribution of F-actin when cells encounter 3D ECM, which indicate the importance of studying cell movement in a 3D microenvironment [2,10].

### Cell morphology and spreading on top and within microposts

**Figure 6:**
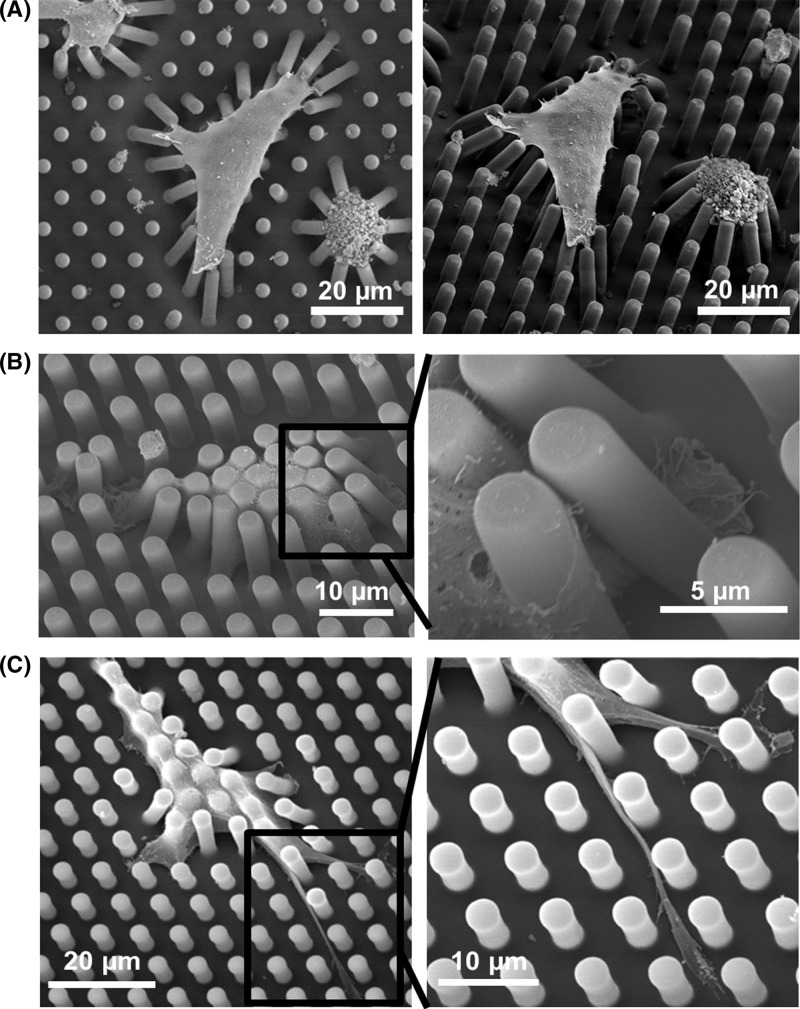
Scanning electron micrographs showing cell spreading on micropost platforms (**A**) FN printed on top, (**B**) FN coated all over and 3 μm spacing, and (**C**) FN coated all over and 5 μm spacing.

To study how cells extend lamellipodia or filapodia during cell migration on top or between microposts, SEM was taken. As shown in Figure 6A, cells spread and only contacted the top surface of the microposts when FN was selectively coated on top. The cells interacted and exerted traction force on top of the microposts. The microposts in leading and trailing regions of the cells were bent toward the center of the cells [25].

When cells were seeded on microposts with FN coated all over, cells spread in between microposts as shown in Figure 6B,C. The cellular body wrapped all around the microposts from top to bottom and cell filapodia was observed to develop along the sidewalls of the microposts. Although the protrusions of cellular filopodia were observed in between the microposts with 3 μm spacing, as shown in Figure 6B, the polarized bundle of lamellipodia was not observed. However, cells had protruded filapodia and their lamellipodia was polarized between microposts with 5 μm spacing, as shown in Figure 6C. From the results of cell nucleus deformation, cytoskeletal development, and cell morphology, cell migration behavior was found to be very different between moving on top and moving in between microposts.

### Cell migration on micropost arrays with various degrees of confinement

During *in vivo* cell migration, cells making contact with ECM consist of different dimensions and confinement. For example, activated dendric cells could squeeze between epidermal cells and reach the lymph vessel [16]. Cell migration not only could be controlled by chemotaxis, but also be modified by cell–matrix interactions. Hence it is essential to acquire insights into cell migration within 3D matrix and various degrees of confinement.

Cell trajectories and speed were compared between cell migration on top of microposts and in between microposts with FN coated all over. To study the confinement created by sidewalls of the microposts with FN coated all over, the spacing between the microposts was changed from 3 to 5 μm. Additional confinement was also provided by adding covers on top of the micropost platforms. Supplementary Movie S1a shows the dynamic changes in the cell morphology during 2D cell migration on top of the microposts. The cells had polarized leading regions and retracted trailing regions during cell migration. This was different from cell migration in between microposts. Although cells constantly extended the filapodia, a stable development of lamellipodia was not observed during cell migration in between the microposts with 3 μm spacing, as shown in Supplementary Movie S1b. Hence the cells had perturbations, but not an overall movement and were trapped in between 3 μm spacing microposts. Similar cell movement was observed for cell migration between microposts with 4 μm spacing, as shown in Supplementary Movie S1c. As the spacing between the microposts was increased to 5 μm, the locomotion of the cell was observed, as shown in Supplementary Movie S1d. In this case, the small bundles of filapodia could protrude between the microposts and formed lamellipodia. Cells were able to squeeze and moved through microposts. As a cover added at 20 μm above the microposts, the cell migration trajectory was similar to cell migration without the cover, as shown in Supplementary Movie S2a. However, when the distance between the microposts and the cover was reduced to 10 μm, the cells showed a much faster migration, as shown in Supplementary Movie S2b. During this cell migration, the cell position changed rapidly in the horizontal and vertical planes, indicating that the cell could contact the top of the microposts and the cover above.

**Figure 7:**
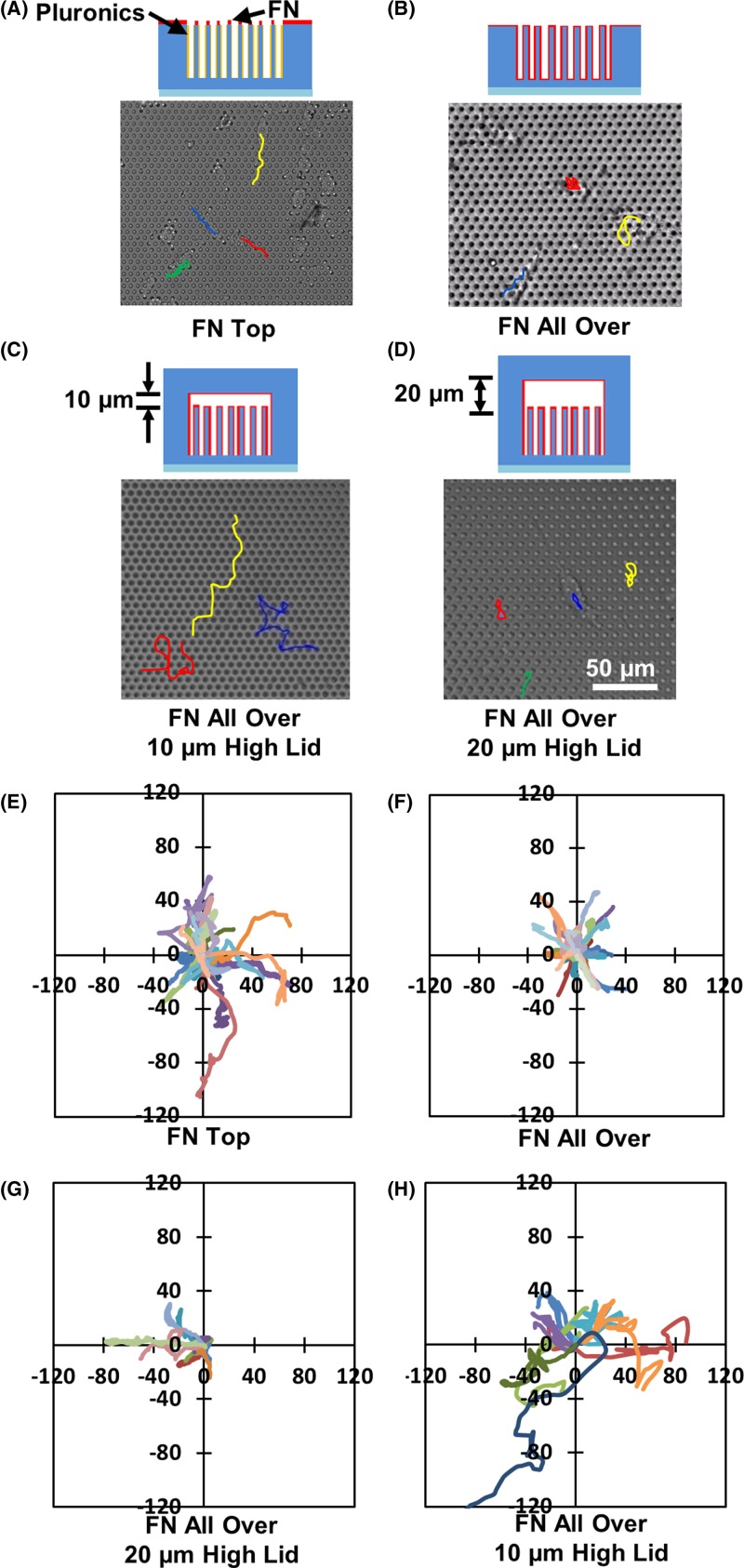
Cell migration trajectories on top of microposts, in between micropost arrays, and inside micropost platforms with covers Cell migration tracks on microposts with (**A**) FN printed on top, (**B**) FN coated all over, covers at (**C**) 10 μm and (**D**) 20 μm above microposts. Microposts were 3 μm in dia. and 5 μm in spacing. Trajectories of cells on microposts with (**E**) FN coated on top, (**F**) FN coated all over, covers at (**G**) 10 μm and (**H**) 20 μm above microposts.

To analyze the cell migration on different platforms, cell migration trajectories were studied. As shown in Figure 7A–H, the cell migration trajectories were tracked for micropost platforms with different degrees of confinement over 6 h. With FN coated on top of the microposts, cells migrated on the top surface with no directional preference. Hence cells had random cell trajectories, as shown in Figure 7A,E. When cells were trapped in between microposts, they had limited movements, as shown in Figure 7B,F. Although cells could protrude the lamellipodia and become elongated when they migrated between microposts with 5 μm spacing, the lower migration speed may be caused by the difficulty for cell deformation. The actin-filament distributions and time-lapse movies also indicated a significant cell nucleus distortion around the microposts. Therefore, cells moved in shorter ranges and their migration direction followed the paths in between the microposts. Figure 7D,H showed the trajectories of cells in confined space with a cover 20 μm above the microposts. In this case, cell trajectories were similar to cell migration in between 5 μm spacing microposts without cover. The cell movement was also constrained by the sidewalls of microposts. When the distance between the cover and the microposts was decreased to 10 μm, cells migrated faster with larger range compared with cell movements on other platforms, as shown in Figure 7C,G.

**Figure 8:**
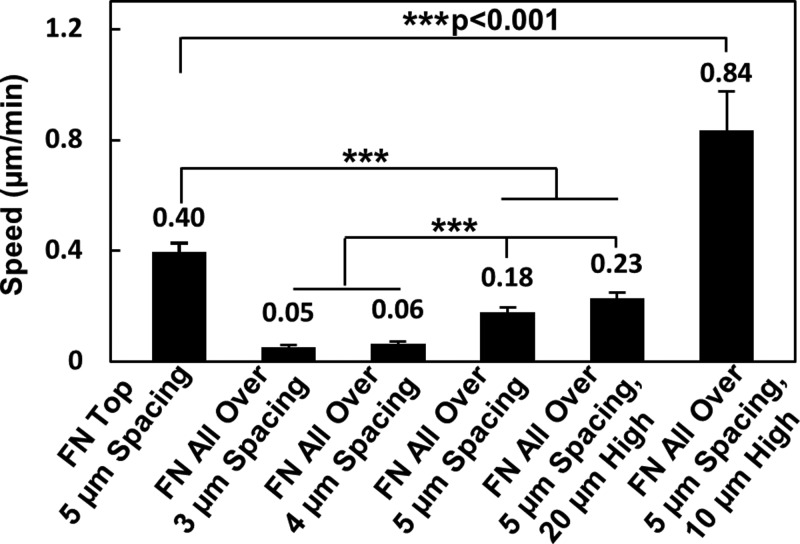
Cell migration speed on micropost platforms Cell migration speed on platforms with FN coated on top and 5 μm spacing and FN coated all over and 3, 4, and 5 μm spacing, and covers at 10 and 20 μm above microposts. Statistical significance was calculated using Student’s *t* test (***P<0.001).

The average cell migration speed on different platforms was shown in Figure 8. Compared with cell migration on top of the microposts with speed of 0.40 ± 0.03 μm/min, cell migration in between microposts had much lower speed. The sidewalls of the microposts constrained cell migration by limiting the elongation of lamellipodia and trapping the cell nuclei. Cells barely moved in between microposts with 3 and 4 μm spacing and the cell migration speed was 0.05 ± 0.01 and 0.06 ± 0.01 μm/min, respectively. When the distance between the microposts was increased to 5 μm, an increased cell speed of 0.18 ± 0.02 μm/min was observed. With larger separation between microposts, cells could have protruded lamellipodia and became elongated. Moreover, the interactions between the actin filament and microposts may enable cells to squeeze through tight space. These results are comparable with cell migration within fibrous collagen matrix, showing no cell movement when cell nucleus was deformed to less than 3 μm [12]. As a cover was added to form a confined space of 20 μm above the micropost arrays, the cell speed of 0.23 ± 0.02 μm/min was similar to cell migration without cover. The cell migration speed increased significantly to 0.84 ± 0.14 μm/min when the cover was 10 μm above the microposts. The time-lapsed images showed that the cells could contact with the microposts and the top cover during cell migration with a faster migration speed. The average cell speed was analyzed during cell migration on top of microposts with different spacings, as shown in Supplementary Figure S2. When FN was coated on top and spacing was 3, 4, and 5 μm, cell contacted top of microposts and migrated at speed of 0.44 ± 0.06, 0.43 ± 0.07, and 0.40 ± 0.03 μm/min, respectively. There was no significant difference for cell migration speed on top of microposts with different spacings. Hence, density of micropost array did not significantly influence cell migration speed when cells moved on top of microposts. However, cell migration slowed down when cells were squeezed in between microposts.

**Figure 9:**
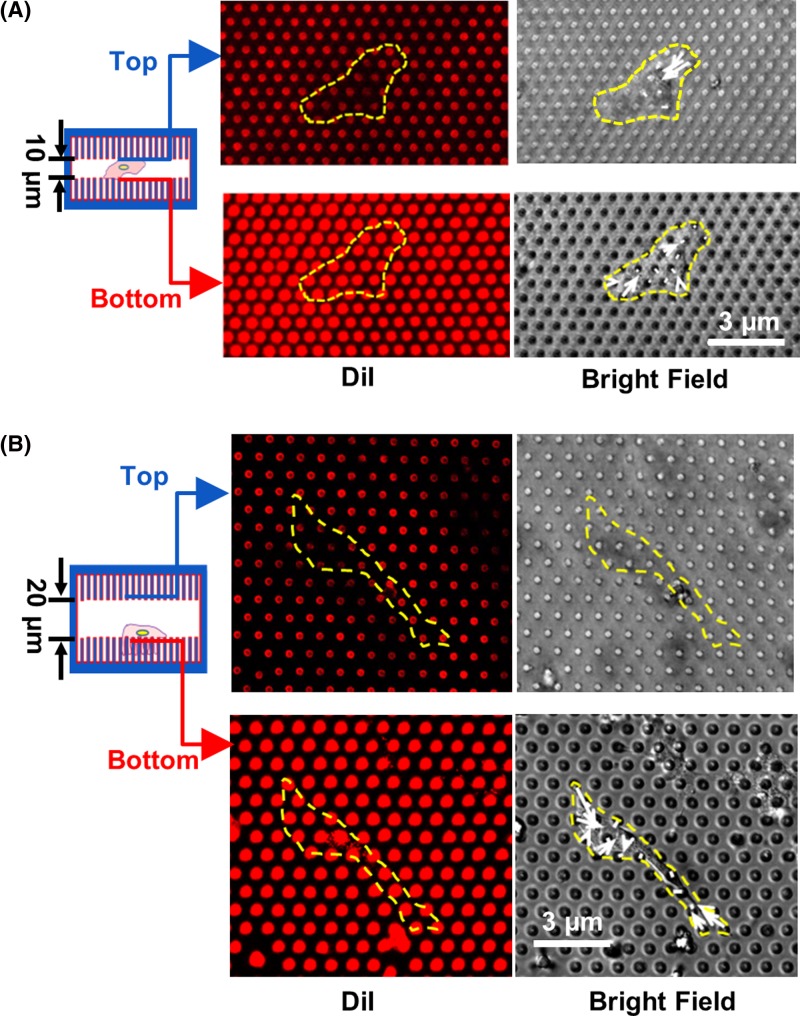
Cellular contacts on top and bottom microposts during cell migration Bending of microposts during cell migration in confined platforms with microposts on both top and bottom surfaces with covers (**A**) 10 and (**B**) 20 μm above microposts.

In order to study cell contacts in confined space, the top cover was replaced by a cover with micropost arrays that were 3 μm in dia. and 5 μm in spacing, the same as the micropost arrays in the bottom. When the distance between the top and bottom micropost arrays was 20 μm, the bending of microposts was only observed at the bottom microposts, as shown in Figure 9B. Hence, the cells mostly adhered and generated traction force on the microposts at the bottom. When separation between the top and bottom micropost arrays was 10 μm, cells contacted both the top and bottom microposts. Therefore, cell traction force was generated on both the top and bottom microposts, as shown in Figure 9A. In this case, cells migrated at higher speed in between the top and bottom microposts. These results are in agreement with previously reported data showing the maximum cell speed when the pore size of confinement was comparable with the cell body [12].

When FN was coated on top, cells extended filapodia on the top surface of microposts, as shown in Supplementary Figure S3. Cell traction force was generated on top of microposts and cell nucleus was not distorted [35]. Hence, cells generated uniform meshwork of actin filament across cellular body. When FN was coated all over, cells could spread in between microposts within 1 h of seeding [20]. During the initial cell spreading along the sidewalls of microposts, cells could migrate toward the bottom of microposts as the lower section of microposts was stiffer than the top [38]. After seeding for 6 h, majority of the cells contacted the bottom and spread near lower part of microposts. As the spacing between adjacent microposts was smaller than the size of cell nucleus, cells were trapped in between microposts [3]. When the spacing between microposts increased from 3 to 5 μm, cell nucleus was less distorted and F-actin was detected around sidewalls of microposts. Cells generated force along sidewalls of micropots to promote actin polymerization between sidewalls [35]. However, the cell movement was limited in between microposts. When a 10-μm cover was placed above microposts, cells could contact the top surface and the cells were not trapped in between microposts. As cell nucleus was not distorted, the generation of cell traction force at both the top cover and the bottom resulted in a higher migration speed [22].

Confined spaces are often encountered by cells found *in vivo* that may be similar to cells being squeezed by nearby microposts [39]. The locomotion of cells inside confined matrix with pore size smaller than cell nucleus is related to many cellular processes, including immune cell response and cancer metastasis [5,6]. When cells were seeded within a collagen matrix with various porosities, cells barely moved when pore size was less than 3 μm, while the maximum cell speed was observed when the pore size was comparable with the cell body [12]. When FN was coated all over, cells spread toward lower part of microposts, similar to previous report [20]. However, the investigation of cell migration under various degrees of confinements was limited. Cell migration in 3–5 μm spaced microposts integrated with a cover at 10–20 μm above was studied for the first time. When a top cover was added and the separation was 10 μm, cells could adhere and generate traction force on the microposts at both top and bottom micropost layers. Cell migration speed and trajectory under various physical confinements were compared. The present study will lead to a better understanding of cell migration in 3D ECM when cells are in contact with platforms with precisely engineered pore sizes and coating conditions in the micropost array.

## Conclusion

Despite extensive studies were conducted for cell migration on 2D surfaces, the understanding of cell–ECM interactions in 3D microenvironment is still limited. As many cellular processes involve cell migration through narrow constrictions *in vivo*, such as capillaries and dense ECM of collagen, the investigation of cell migration within confined 3D ECM is essential.

In the present study, MC3T3-E1 osteoblast cell migration within 3D microenvironments with various degrees of confinement was investigated. To acquire insights into cell migration within confined structures, the fluorescent distributions of cell nucleus, actin filament, and vinculin were investigated. The morphologies of cell spreading within 3D matrix were obtained using SEM. The cell migration trajectories and speed were compared during cell migration on micropost platforms with various confinements. To create the 3D platforms, covers were placed at different separation distance above the micropost arrays.

**Figure 10:**
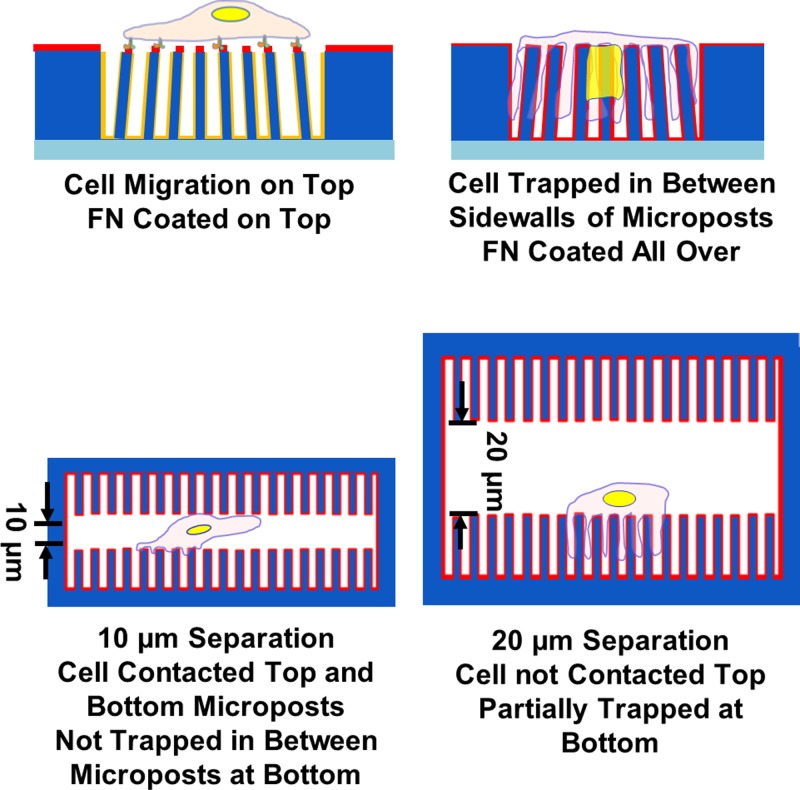
Schematics of cell migration on micropost platforms with various degrees of confinements

The protein coating conditions determined the cell spreading and location on the micropost platforms. The schematics of cell migration on platforms with various confinements are shown in Figure 10. FN coated on top kept cells on top while FN coated all over trapped the cells between the microposts. Compared with rounded cell nuclei during cell spreading on top of the microposts, the cell nuclei were deformed between microposts for cells trapped in between microposts with FN coated all over. Prominent actin filaments were generated at cell periphery and around the sidewalls of microposts when cells were located in between the microposts. The SEM showed the formation of lamellipodia between microposts when post-to-post spacing was 5 μm. During 2D cell migration, the cell locomotion was initiated by extension and retraction of cell leading and trailing regions. When cells moved in between microposts, they had to squeeze between the microposts with slower speed. With the addition of a second set of micropost arrays at 10 μm above the microposts, the cells moved at higher speed and no longer trapped in between the microposts. These results showed cell migration and cell–ECM interactions within confined microenvironment, which is important to understand cell migration in confined spaces.

## Supporting information

**Supplementary Figures:** 

**Supplementary Material:** 

**Supplementary Material:** 

**Supplementary Material:** 

**Supplementary Material:** 

**Supplementary Material:** 

**Supplementary Material:** 

## Abbreviations

dia.: diameter
DiI: 1,10-dioleyl-3,3,30,30-tetramethylindocarbocyanine methanesulphonate
DMEM: Dulbecco’s modified Eagle’s medium
ECM: extracellular matrix
FN: fibronectin
LINC: linker of the nucleoskeleton and cytoskeleton complex
PDMS: polydimethylsiloxane
PFA: paraformaldehyde
SEM: scanning electron microscope
