# Supplementary figures and images for: Cell migration on microposts with surface coating and confinement
Source: Biosci Rep. 2019 Feb 19;39(2):BSR20181596. doi: 10.1042/BSR20181596 (PMC6379512; doi:10.1042/BSR20181596)

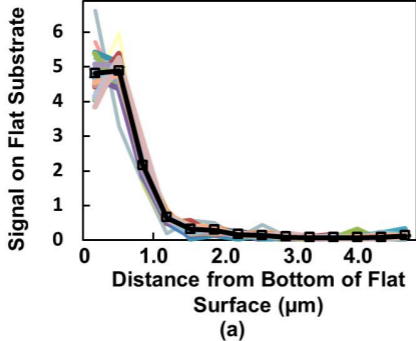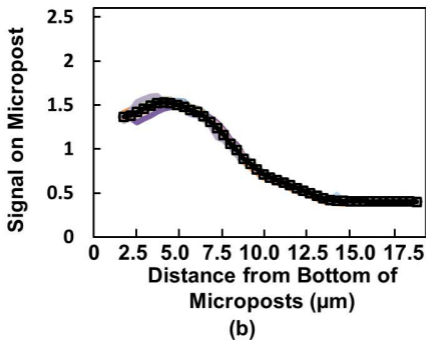

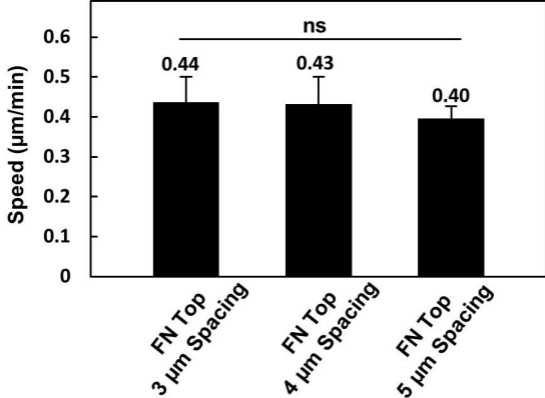

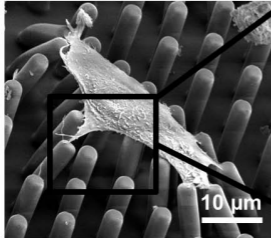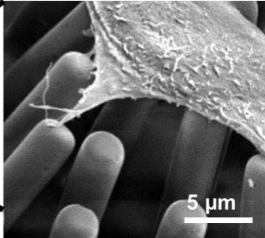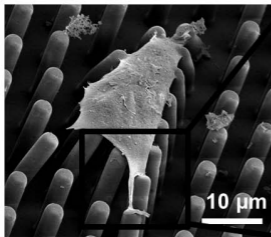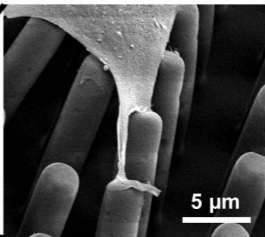

Supplement: Supplementary file 1 [file bsr-39-bsr20181596_Supp1.pdf]
